# Aberrant X chromosomal rearrangement through multi‐step template switching during sister chromatid formation in a patient with severe hemophilia A

**DOI:** 10.1002/mgg3.1390

**Published:** 2020-07-05

**Authors:** Mahiru Tokoro, Shogo Tamura, Nobuaki Suzuki, Misaki Kakihara, Yuna Hattori, Koya Odaira, Sachiko Suzuki, Akira Takagi, Akira Katsumi, Fumihiko Hayakawa, Shuichi Okamoto, Atsuo Suzuki, Takeshi Kanematsu, Tadashi Matsushita, Tetsuhito Kojima

**Affiliations:** ^1^ Department of Pathophysiological Laboratory Sciences Nagoya University Graduate School of Medicine Nagoya Japan; ^2^ Department of Transfusion Medicine Nagoya University Hospital Nagoya Japan; ^3^ Shubun University Ichinomiya Japan; ^4^ Department of Transfusion Medicine National Center for Geriatrics and Gerontology Obu City Japan; ^5^ Department of Hematology and Oncology Nagoya University Graduate School of Medicine Nagoya Japan; ^6^ Department of Clinical Laboratory Nagoya University Hospital Nagoya Japan

**Keywords:** chromosomal rearrangement, *F8*, FoSTeS/MMBIR, hemophilia A, homologous recombination, inversion

## Abstract

**Background:**

Hemophilia A (HA) is an X‐linked recessive bleeding disorder caused by pathogenic variants of the coagulation factor VIII gene (*F8*). Half of the patients with severe HA have a recurrent inversion in the X chromosome, that is, *F8* intron 22 or intron 1 inversion. Here, we characterized an abnormal *F8* due to atypical complex X chromosome rearrangements in a Japanese patient with severe HA.

**Methods:**

Recurrent *F8* inversions were tested with inverse shifting‐PCR. The genomic structure was investigated using PCR‐based direct sequencing or quantitative PCR.

**Results:**

The proband's X chromosome had a 119.5 kb insertion, a reverse duplex of an extragenic sequence on the *F8* telomere region into the *F8* intron 1 with two breakpoints. The telomeric breakpoint was a joining from the *F8* intron 1 to the inverted *FUNDC2* via a two‐base microhomology, and the centromeric breakpoint was a recombination between *F8* intron 1 homologous sequences. The rearrangement mechanism was suggested as a multi‐step rearrangement with template switching such as fork stalling and template switching (FoSTeS)/microhomology‐mediated break‐induced replication (MMBIR) and/or homologous sequence‐associated recombination during a sister chromatid formation.

**Conclusion:**

We identified the aberrant X chromosome with a split *F8* due to a multi‐step rearrangement in a patient with severe HA.

## Graphical abstract

Hemophilia A (HA) is an X‐linked recessive bleeding disorder caused by mutations in the coagulation factor VIII gene (*F8*). We identified the aberrant chromosome X with a split *F8* due to a multi‐step rearrangement in a severe HA patient. The rearrangement mechanism was suggested as a multi‐step rearrangement with template switching such as FoSTeS/MMBIR and/or homologous sequence associating recombination during a sister chromatid formation.

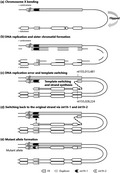

## 1 INTRODUCTION

Hemophilia A (HA) is an X‐linked recessive bleeding disorder caused by pathogenic variants of the coagulation factor VIII gene (*F8*, OMIM: 300841). Based on the coagulation factor VIII (FVIII) activity (FVIII:C), HA is classified into three phenotypes: severe (FVIII:C <1 IU/dL), moderate (1 ≤ FVIII:C <5 IU/dL), and mild (5 ≤ FVIII:C <40 IU/dL). *F8* consists of 26 exons and 25 introns spanning 186 kb at a long arm of the X chromosome (Xq28; Lannoy & Hermans, 2016). Thus far, 3,052 of the various *F8* pathogenic variants, including unique structural variations such as a large deletion or duplication, have been reported in the *F8* variant database (http://www.factorviii‐db.org/, accessed on 5 April 2020).

Half of the patients with severe HA have a recurrent intra‐chromosomal inversion known as intron 22 inversion (Inv22) caused by nonallelic homologous recombination (NAHR) between homologous sequences: *int22h‐*1 within *F8* intron 22, and either *int22h‐*2 or *int22h‐*3 located at 400 kb telomeric distal from *F8* (Lakich, Kazazian, Antonarakis, & Gitschier, 1993; Naylor, Brinke, Hassock, Green, & Giannelli, 1993). Moreover, the *F8* intron 1 inversion (Inv1) is responsible for the genetic defect in 1%–5% of all severe HA cases. Inv1 is due to the intra‐chromosomal NAHR crossing over two almost identical sequences: *int1h‐*1 in the *F8* intron 1 and *int1h‐*2 located at approximately 126 kb telomeric distal from *F8* (Bagnall, Waseem, Green, & Giannelli, 2002). In addition to the typical *F8* Inv22 or Inv1, several studies have pointed out that some cases of severe HA carry an unusual *F8* structural abnormality that may associate with inversions. The *int22h*s‐associated recombination between *F8* and neighbor genes causes large deletions or duplications (Chen et al., 2017; Fujita et al., 2012). Moreover, several reports suggested that *int1h*s is also involved in large duplications and/or deletions within a telomeric region of the X chromosome. (Pio, Oliveira, Soares, & Rezende, 2011; Sanna et al., 2013; Sukarova Stefanovska, Dejanova, Tchakarova, Petkov, & Efremov, 2008; You et al., 2014). However, to our knowledge, the mechanism by which those complex *F8* rearrangements occur has not been thoroughly investigated.

In this study, we analyzed the *F8* in a Japanese patient with severe HA and characterized the abnormal X chromosomal structure, NC_000023.11:g.155007148_155013478delins155028224_155147780inv, that was an insertion of a large duplicon derived from the *F8* extragenic alignment into the *F8* intron 1. We thus proposed a hypothetical mechanism by which the aberrant X chromosome was constructed through multi‐step rearrangement with replicative DNA repair models such as the fork stalling and template switching (FoSTeS)/microhomology‐mediated break‐induced replication (MMBIR) and homologous recombination (HR) during a sister chromatid formation.

## 2 MATERIALS AND METHODS

### 2.1 Patient and DNA sample

**Figure 1:**
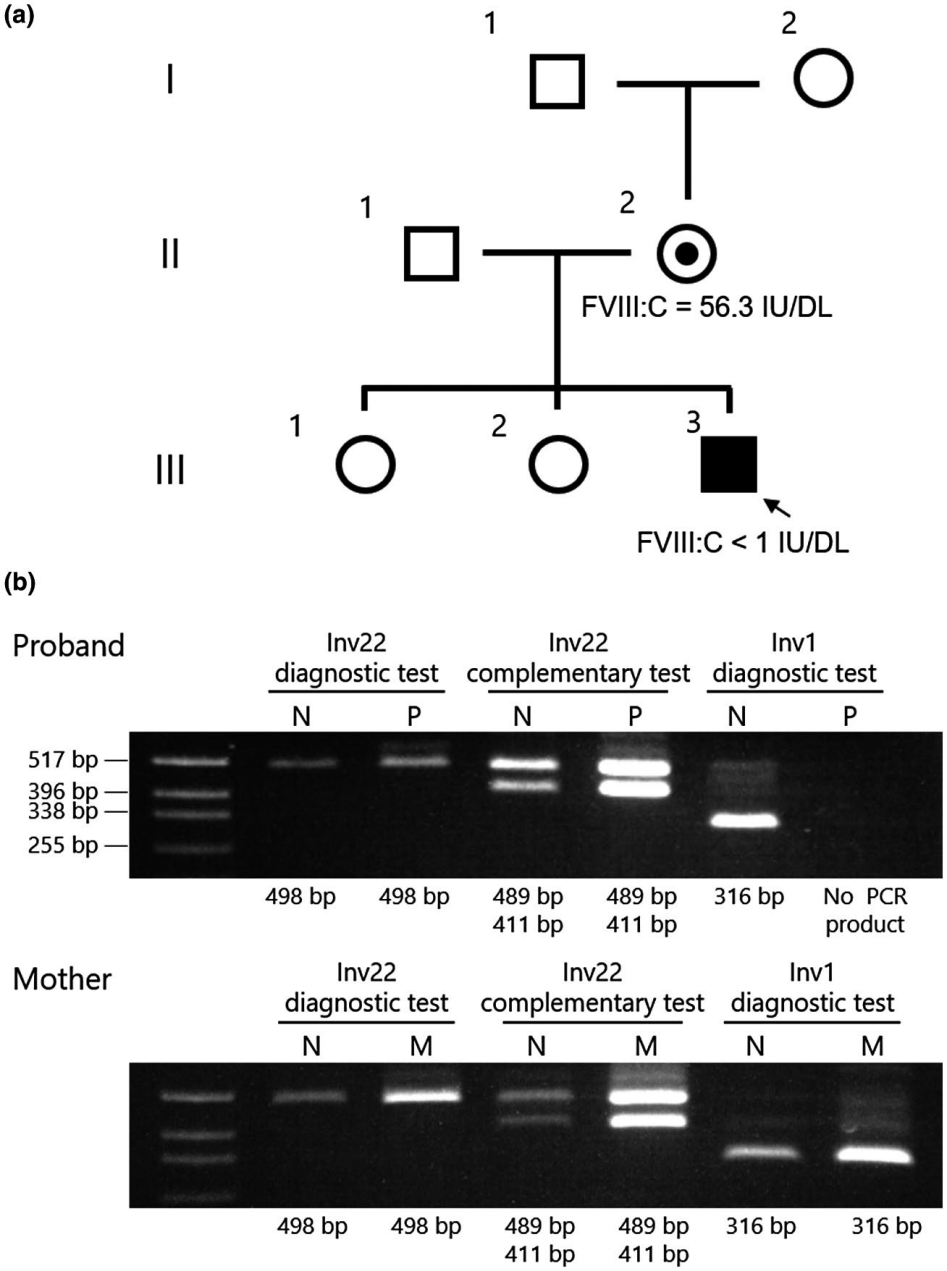
Inverse shifting‐PCR (IS‐PCR) suggested a structural abnormality in the proband's *F8* intron 1. (a) Family pedigree. Proband (III‐3) is indicated by an arrow. The proband's mother (II‐2) showed reduced FVIII activity (FVIII:C = 56.3 IU/dL) and had bleeding symptoms at the time of surgery. The proband's father (II‐1) and maternal grandparents (I‐1 and I‐2) did not have any bleeding tendency. (b) Recurrent inversion analysis with IS‐PCR. N, normal male control; P, proband; M, mother. In Inv1 diagnostic test, the proband's PCR product was not observed (expected band sizes are normal: 316 bp and Inv1: 281 bp, respectively). Other tests showed a pattern similar to that in a normal control (Inv22 diagnostic test: 498 bp, Inv22 complementary test: 489 and 411 bp)

The proband was a 6‐month‐old Japanese boy affected by severe HA (FVIII:C <1 IU/dL, Figure 1a III‐3). The proband's mother showed reduced FVIII activity (FVIII:C = 56.3 IU/dL, Figure 1a II‐2) and had bleeding symptoms at the time of surgery. The proband's father and maternal grandparents did not have any bleeding tendency. The FVIII antigen level (FVIII:Ag) was measured using the VisuLize Factor VIII ELISA Kit (Affinity Biologicals Inc., Ancaster, Canada) and the FVIII:Ag in the proband's plasma was lower than the limit of detection (<0.8%). After informed consent was obtained from the parents, genomic DNA (gDNA) was extracted from peripheral blood cells as described previously (Kojima et al., 1987). This study was approved by the Ethics Committee of Nagoya University School of Medicine (Identification number: 2015‐0391).

### 2.2 *F8* intron 22 and intron 1 inversions analysis by inverse shifting‐PCR (IS‐PCR)

IS‐PCR was performed as previously described (Rossetti, Radic, Larripa, & De Brasi, 2008) with minor modifications. About 2 μg of gDNA was digested with 15 U of *Bcl*I (New England Biolabs) for 3 hr in a total reaction volume of 50 μL as per the manufacturer's instruction. The restriction enzyme was inactivated through a phenol/chloroform/isoamyl alcohol extraction followed by ethanol precipitation, and gDNA was dissolved in 4 μL of TE buffer (10‐mM Tris with 1‐mM EDTA, pH 8.0). The digested gDNA samples were self‐ligated using Ligation high ver. 2 (Toyobo Co., Ltd.) for 6 hr at 16°C in a reaction volume of 8 μL (4 μL of the digested gDNA was mixed with an equal amount of Ligation high ver. 2). After ethanol precipitation, ligated samples were resolved with 30 μL of TE buffer. PCR was performed in a reaction volume of 20 μL containing 1 μL of the ligated gDNA, 0.5 μM of each primer, 0.4 U of KOD FX DNA polymerase (Toyobo), and additional PCR reaction reagents for the KOD FX. Primer sets for IS‐PCR are listed in Table S1. Thermal cycling was performed using the step‐down procedure preceded by a hot‐starting step as follows: 94°C for 2 min; five cycles of denaturation at 98°C for 10 s, annealing at 68°C for 30 s, and extension at 68°C for 35 s; then five cycles of denaturation at 98°C for 10 s, annealing at 66°C for 30 s, and extension at 68°C for 35 s; further 25 cycles of denaturation at 98°C for 10 s, annealing at 65°C for 30 s, and extension at 68°C for 35 s; followed by a final extension at 68°C for 2 min. PCR products were analyzed using electrophoresis on a 1.5% agarose gel and stained with 1 μg/mL of ethidium bromide.

### 2.3 *F8* DNA sequencing

All 26 exons, including exon‐intron junctions, promoter region, and untranslated regions, were amplified via PCR using originally designed gene‐specific primers (Table S2). The PCR was performed in reaction volumes of 20 μL containing 80 ng of gDNA, 0.5 μM of each primer, and 10 μL of AmpliTaq Gold 360 Master Mix (Thermo Fisher Scientific Inc.). The cycling conditions were: 30 cycles of denaturation at 95°C for 30 s, annealing at 55°C for 30 s, and extension at 72°C for 30 s, preceded by 95°C for 10 min, followed by 72°C for 2 min. PCR products were quantified using electrophoresis on 1.5% agarose gels. The DNA sequencing reaction was conducted with the BigDye Terminator v1.1 Cycle Sequencing Kit (Thermo Fisher Scientific Inc.) according to the manufacturer's instructions. Samples were analyzed with the ABI PRISM 310 Genetic Analyzer or ABI PRISM 3130 Genetic Analyzer (Thermo Fisher Scientific Inc.). In this study, the nucleotide position at X chromosome was referred in GenBank: NC_000023.11.

### 2.4 Multiplex ligation‐dependent probe amplification (MLPA) analysis for *F8*

MLPA was performed using the SALSA MLPA P178 *F8* probemix (MRC‐Holland) according to the manufacturer's instructions. DNA fragments were analyzed with the ABI PRISM 310 Genetic Analyzer. The relative quantity of the patient *F8* was evaluated in comparison with that of the pooled Japanese healthy male gDNA control.

### 2.5 Amplification of the *F8* intron 1

**Figure 2:**
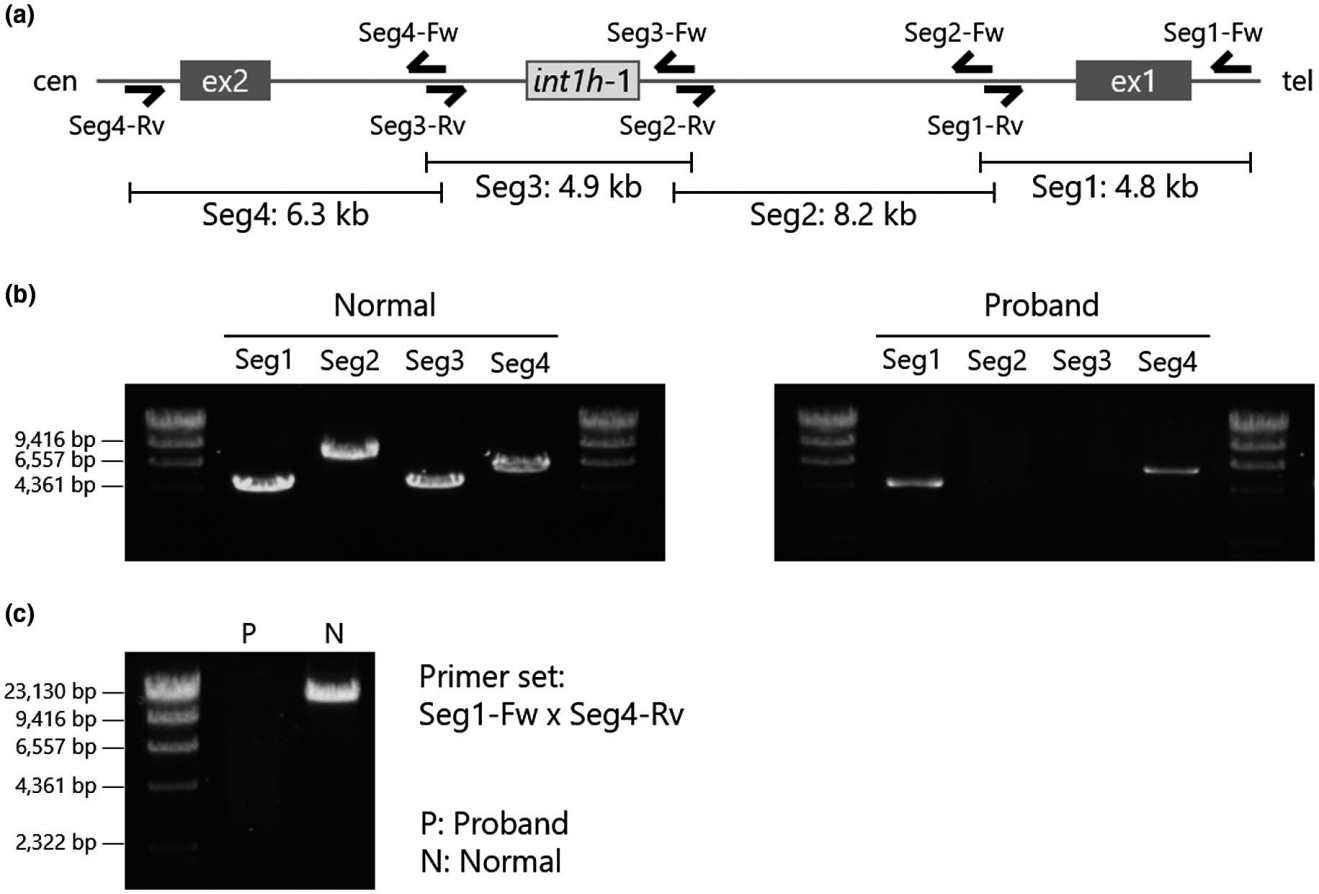
PCR mapping in *F8* intron 1. (a) Primer design of mapping PCR in *F8* intron 1. *F8* intron 1 is divided into four segments, Seg 1 (4.8 kb), Seg 2 (8.2 kb), Seg 3 (4.9 kb), and Seg 4 (6.3 kb). (b) Results of *F8* intron 1 mapping in normal male individual (left) and proband (right). Segments 2 and 3 were not detected in the proband's gDNA. (c) Full‐length PCR amplification of *F8* intron 1. Long‐range PCR successfully amplified the product in the gDNA of normal male subject (N), while no amplicon was detected in the proband's gDNA (P)

To analyze the *F8* intron 1, a PCR amplicon was designed and divided into four segments (Figure 2a). The *F8* intron 1 specific primer sets are listed in Table S3. PCR was performed in reaction volumes of 20 μL containing 80 ng of circularized DNA, 0.3 μM of each primer, 0.4 U of KOD FX Neo DNA polymerase (Toyobo co., Ltd.), and additional PCR reaction reagents for the KOD FX Neo. The cycling conditions were: 25 cycles of denaturation at 98°C for 30 s, annealing at 63°C for 30 s, and extension at 68°C for 5 min, preceded by 98°C for 2 min, followed by 68°C for 7 min. The PCR products were analyzed using electrophoresis on a 1% agarose gel. To detect the breakpoint of large deletions within intron 1, the *F8* intron 1 was entirely amplified with the primers seg1‐Fw and seg4‐Rv (Table S3) and the KOD FX Neo DNA polymerase. The PCR cycling conditions were as described above with a modification of the extension time to 14 min.

### 2.6 Inverse PCR

**Figure 3:**
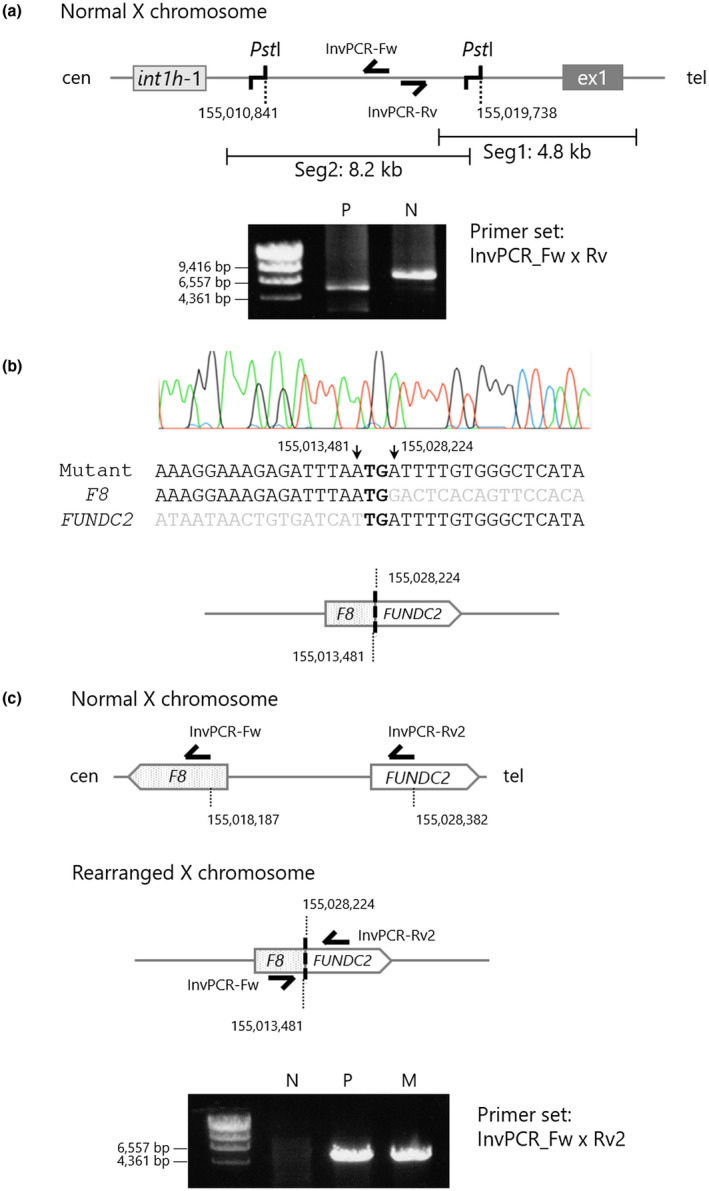
Identification of a telomeric breakpoint in the proband's *F8* intron 1. (a) Design of inverse PCR with *Pst*I site within *F8* intron 1 segments 1–2 (upper) and PCR results in a normal male subject (N) and the proband (P; lower). *Pst*I‐inverse PCR suggested that the proband's *F8* intron 1 had a structure abnormality. (b) Direct sequencing of the abnormal inverse PCR product derived from the proband's gDNA. The electropherogram of a breakpoint connecting *F8* intron 1 (NC_000023.11 nt155,013,481) to inverted *FUNDC2* (NC_000023.11 nt155,028,224) via two bases of microhomology (upper) and its speculated structural diagram (lower). (c) Mutant‐specific PCR with primer set, InvPCR‐Fw and InvPCR‐Rv2. Design of the mutant‐specific PCR (upper) and the PCR results in a normal male subject, the proband, and the proband's mother. N: normal male subject, P: proband, M: proband's mother

**Figure 4:**
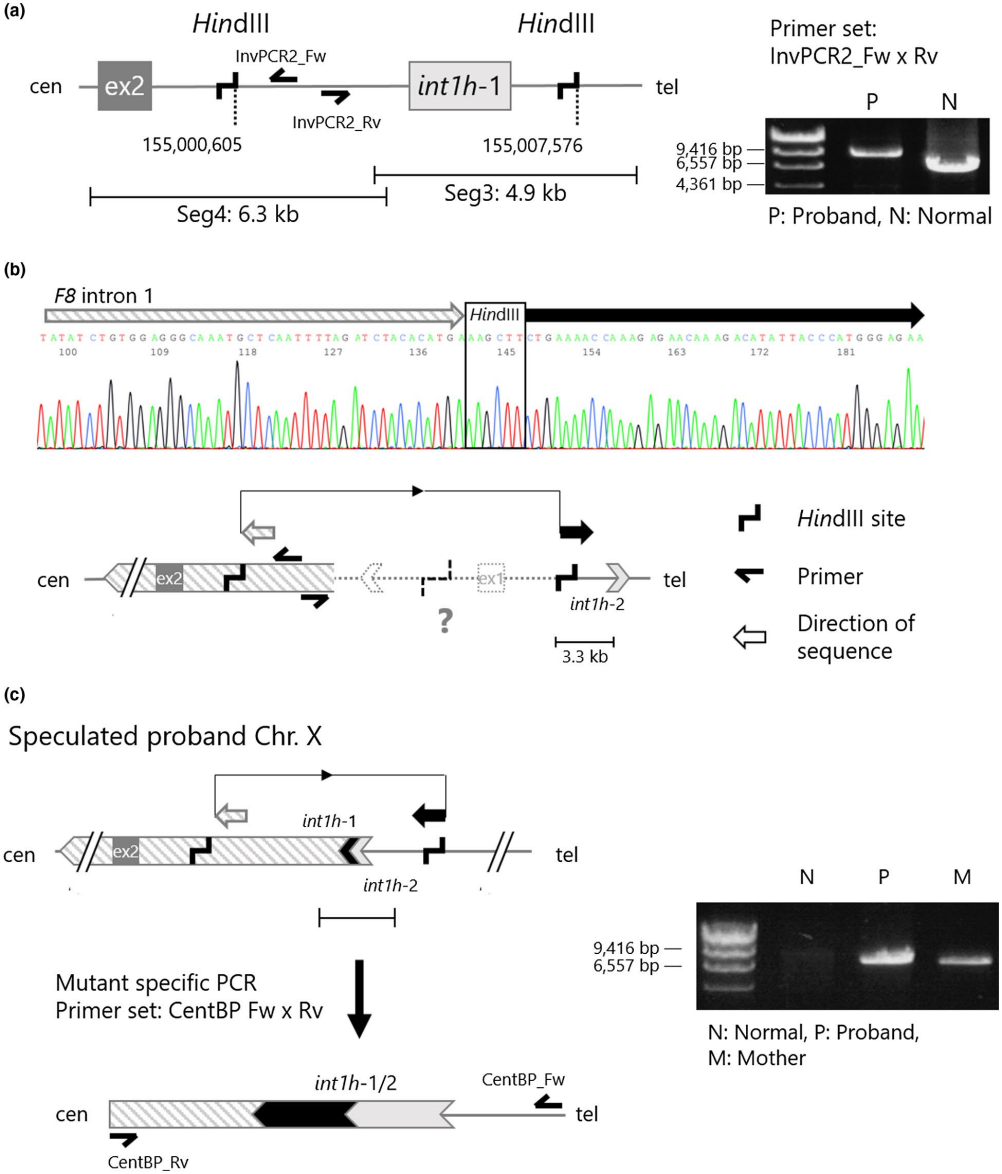
Identification of a centromeric breakpoint in the proband's *F8* intron 1. (a) Design of inverse PCR with *Hin*dIII site within *F8* intron 1 segments 3–4 (left), and the PCR results in a normal male subject (N) and the proband (P; right). *Hin*dIII‐inverse PCR suggested that the proband's *F8* intron 1 had another structure abnormality at the centromeric side. (b) Direct sequencing result of the abnormal inverse PCR product derived from the proband's gDNA. The structural diagram explains a sequence direction detected in the direct sequencing. (c) Mutant‐specific PCR with primer set, CentBP_Fw and CentBP_Rv. Design of the mutant‐specific PCR (left) and the PCR results in a normal male subject, the proband, and the proband's mother (right). N: normal male subject, P: proband, M: proband's mother

Inverse PCR was used to investigate the genomic structure of the mutated *F8*. Primers for inverse PCR were designed in opposite orientation in the known regions (Table S3, Figures 3a and 4a). gDNA was digested with *Pst*I (Takara Bio Inc.) or *Hin*dIII (New England Biolabs Japan Inc.) at the appropriate temperature, and fragments were circularized with the Ligation high ver. 2. After ethanol precipitation, the circularized gDNA was resolved with 20 μL of TE buffer. PCR was performed in a total volume of 20 μL containing 1 μL of circularized gDNA, 0.4 U of the KOD FX DNA polymerase, 0.3 μM of each primer, and additional PCR reagents for the KOD FX DNA polymerase. PCR was performed in a step‐down procedure as follows: five cycles of denaturation at 98°C for 10 s, annealing at 70°C for 30 s, and extension at 70°C for 15 min. Subsequently, the annealing temperature was modified to 68°C for five cycles, 66°C for five cycles, and 64°C for 25 cycles. Cycling was preceded by 94°C for 2 min, followed by a final extension at 68°C for 2 min. The PCR product was analyzed using electrophoresis on 1% agarose gel and purified with the FastGene Gel/PCR Extraction Kit (Nippon Genetics Co., Ltd.). The direct sequencing of variant‐specific PCR products was performed using the BigDye Terminator v1.1 Cycle Sequencing Kit and ABI PRISM 3130 Genetic Analyzer.

### 2.7 Long‐range PCR analysis for the *F8* variant associated with the intron 1 homologous region (*int1h*; Inv1 diagnostic long‐PCR)

In order to investigate the *F8* structure variation involved with *int1h,* including Inv1, we originally designed a specific primer set for *int1h*‐1 and *int1h*‐2 (Table S1 and Figure S1a). PCR was performed in a reaction volume of 20 μL containing 80 ng of gDNA, 0.3 μM of each primer, 0.4 U of KOD FX Neo DNA polymerase (Toyobo co., Ltd.), and additional PCR reaction reagents for the KOD FX Neo. The thermal cycling conditions were 25 cycles of denaturation at 98°C for 30 s, annealing at 68°C for 30 s, and extension at 68°C for 7 min, preceded by 98°C for 2 min, followed by 68°C for 7 min. The products were analyzed using electrophoresis on 0.6% agarose gels.

### 2.8 Quantitative gene mapping in the X chromosome

**Figure 5:**
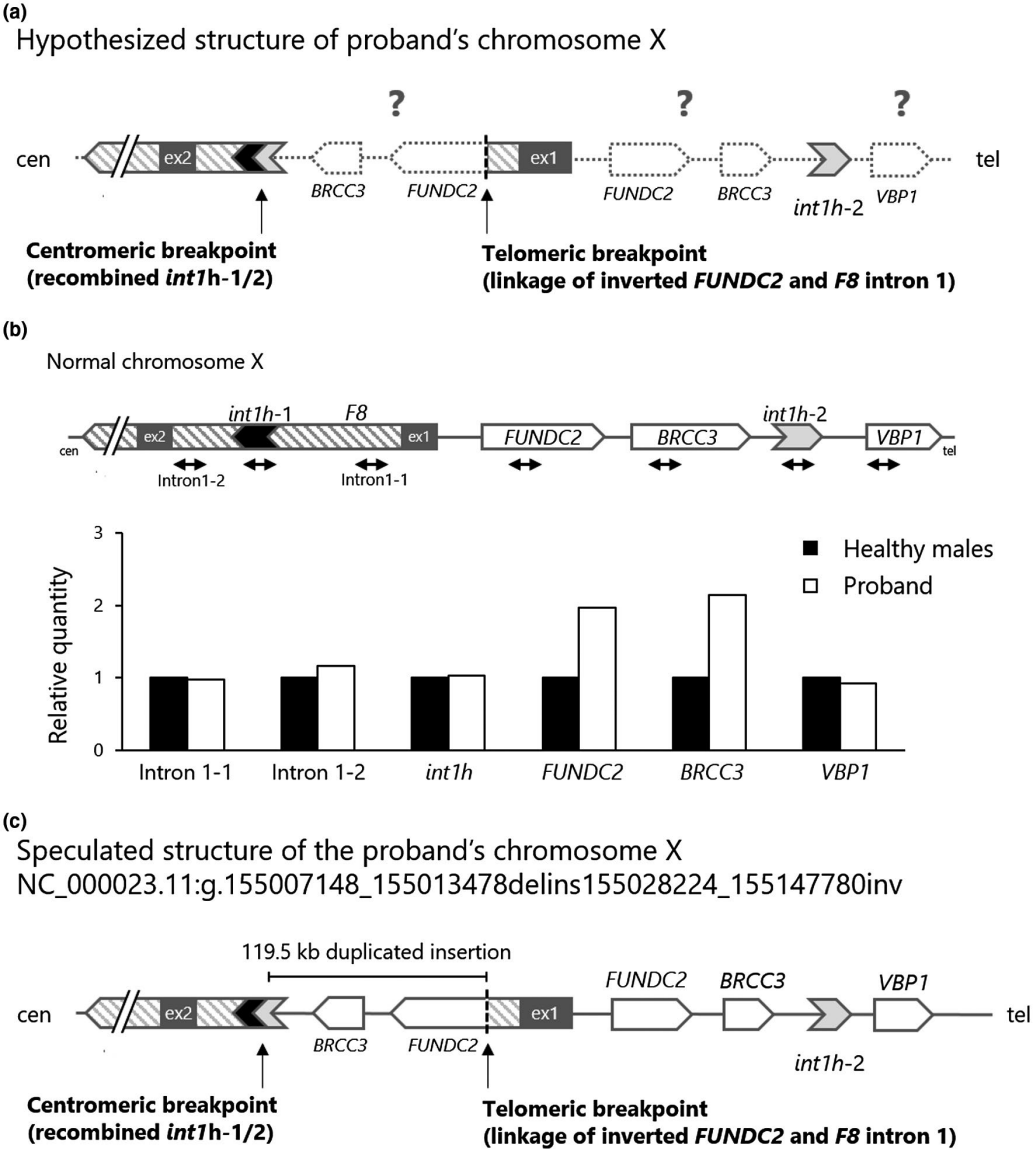
Speculation of the proband's chromosome X structure. (a) Hypothesized structure of proband's chromosome X, based on the observations of two breakpoints and a partial inversion structure. Dotted objects show uninvestigated regions. (b) Quantitative gene mapping analysis. Design of qPCR on the *F8* and its neighboring loci (upper) and the results of copy number variations (lower). Gene dosage was normalized to a gDNA pooled from three normal male individuals. *FUNDC2* and *BRCC3* were assessed as a doubled gene dosage. (c) Speculated structure of the proband's chromosome X (NC_000023.11:g.155007148_155013478delins155028224_155147780inv). The patient *F8* was split with an over 119.5 kb of intra‐chromosomal inverted/duplicated alignment

Quantitative gene mapping was performed using quantitative PCR (qPCR). Specific primer sets were originally designed at neighboring genes of the *F8* (Table S4 and Figure 5b). qPCR was performed in a total volume of 15 μL containing 15 ng of sonicated gDNA, 0.4 μM of each specific primer, and 7.5 μL SYBR Premix Ex Taq II Tli RNaseH Plus (Takara Bio Inc.) The PCR reaction was performed and analyzed with the Thermal Cycler Dice Real Time System II (Takara Bio Inc.).

### 2.9 Reverse transcription (RT)‐PCR analysis of *F8* mRNA in PBMCs

Peripheral blood mononuclear cells (PBMCs) were separated from 5 ml of EDTA anticoagulated whole blood using the Ficoll‐Paque Premium (GE Healthcare UK Ltd.). Total mRNA derived from PBMCs was extracted with the RNeasy Mini Kit (Qiagen K.K.). cDNA was synthesized using the PrimeScript II Reverse Transcriptase with oligo dT primer or random 6 mer primer (Takara Bio Inc.), and PCR amplification was performed in reaction volumes of 20 μL containing 1 μL of cDNA, 0.3 μM of each primer, 0.4 U of KOD FX DNA polymerase (Toyobo co., Ltd.), and additional PCR reaction reagents for the KOD FX. The thermal cycling conditions were 30 cycles of denaturation at 98°C for 30 s, annealing at 60°C for 30 s, and extension at 68°C for 90 s, preceded by 98°C for 2 min, followed by 68°C for 7 min. The products were analyzed using electrophoresis on a 1% agarose gel. We used four sets of primers to amplify the *F8* cDNA according to a previous report with minor modifications (Table S4; El‐Maarri et al., 2005). *GAPDH* was used as the RT‐PCR validating internal control. The PCR product was analyzed using electrophoresis on a 1.2% agarose gel.

## 3 RESULTS

### 3.1 Conventional *F8* variant analysis showed an atypical *F8* inversion in proband's X chromosome

We first performed IS‐PCR to investigate *F8* recurrent inversions, Inv22 and Inv1 (Figure 1b). A pattern of the proband's PCR product was identical to that of a healthy male subject in Inv22 diagnostic and complementary tests. In contrast, the Inv1 diagnostic test did not detect any PCR product in the proband's gDNA. To investigate an exonic deletion or duplication, we quantitatively analyzed a copy number of *F8* exons with MLPA (Figure S2). However, the proband's gDNA did not present any copy number alteration in the *F8* exons. Moreover, no single base substitution, small insertion, or deletion was detected in the *F8* exons and their flanking splice sites. These results suggested that the proband carried a causative structural abnormality in the *F8* intron 1.

### 3.2 PCR mapping analysis showed a complex structure abnormality in the proband's *F8* intron 1

In order to investigate the abnormality of *F8* intron 1, we designed the primer sets by dividing the *F8* intron 1 into four segments (Figure 2a). In a healthy male subject, we detected the products' size as expected: 4.8 kbp, 8.2 kbp, 4.9 kbp, and 6.3 kbp in segments 1, 2, 3, and 4, respectively (Figure 2b left). In the proband, although the products of segments 1 and 4 were clearly detected, those of segments 2 and 3 were not observed (Figure 2b right) suggesting that an unexpected structural abnormality was present in the region of segments 2 to 3. To investigate the structural abnormality in the *F8* intron 1, we amplified the full length of intron 1 using primers Seg1‐Fw and Seg4‐Rv (Figure 2a). However, no PCR product was observed in the patient gDNA (Figure 2c). These results suggested that the proband possessed a gross structural abnormality in the *F8* intron 1. In addition, regarding the other *F8* introns, no other remarkable structural abnormality was observed (Figure S3).

### 3.3 Identification of a telomeric breakpoint in the patient *F8* intron 1

In order to investigate the structural abnormality observed in segments 2 and 3 of the *F8* intron 1, we performed inverse PCR (Figure 3). In a normal *F8* intron 1, *Pst*I sites are present at the positions 3.0 and 11.9 kb from the splice donor site of the *F8* exon 1. The primer set was designed in an opposite orientation within the *Pst*I‐digested fragment (Figure 3a top). In the proband, *Pst*I‐digested gDNA was amplified as an abnormal product with an approximate size of 6 kbp (Figure 3a bottom). Sequencing of the inverse PCR product revealed that the *F8* intron 1 was connected to the inverted *FUNDC2* that was at 4 kb telomeric distal from *F8* on the reference sequence (NC_000023.11). The direct sequencing demonstrated the breakpoint connecting *F8* intron 1 (NC_000023.11 nt155,013,481) to the inverted *FUNDC2* (NC_000023.11 nt155,028,224) via two bases of microhomology (Figure 3b). In order to confirm the unusual structure detected in the proband's X chromosome, we designed a variant‐specific PCR that detected only the rearranged chromosome (Figure 3c). A reverse primer, named InvPCR‐Rv2, was designed in the *FUNDC2* intron 1, which had the same orientation to a counterpart forward primer, InvPCR‐Fw (Figure 3c top and middle). This primer set successfully amplified a product only in the proband and his mother gDNA (Figure 3c bottom).

### 3.4 Identification of a centromeric breakpoint in the patient *F8* intron 1

To further investigate the structure abnormality of the proband's *F8*, we examined segment 4 in the *F8* intron 1 with inverse PCR (Figure 4). In a normal X chromosome, *Hin*dIII sites are present in segments 3 and 4 (positioned at 15.1 kb and 22.1 kb away from the splice donor site of the *F8* exon 1). The primer set was designed at the centromeric side within the *Hin*dIII‐digested fragment (Figure 4a, left). The inverse PCR with the *Hin*dIII‐digested patient gDNA showed an abnormal PCR product (8.5 kbp) as compared to that of a healthy male subject (6.5 kbp; Figure 4a, right). Direct sequencing of the abnormal product showed that segment 4 of the proband *F8* intron 1 was connected to an inverted DNA alignment that was approximately 3.3 kb centromeric away from *int1h‐*2 (Figure 4b). This result suggested an inversion structure of the *F8* intron 1 via *int1h*‐1 and 2.

In order to identify a centromeric breakpoint in the patient *F8* intron 1, we designed a mutant‐specific PCR with a primer set, CentBP_Fw and CentBP_Rv (Figure 4c left). The mutant‐specific PCR successfully amplified a product from the proband's and mother's gDNA, but not from a healthy subject (Figure 4c right). The direct sequencing denoted that the mutant‐specific amplicon contained a DNA alignment of *int1h*‐1 and *int1h*‐2 (Figure S4). These observations indicated that the proband's X chromosome had an inversion structure with a recombination between *F8 int1h*‐1 and 2.

### 3.5 Putative proband's X chromosome structure

To further investigate the structure of the proband's X chromosome, we performed *F8* Inv1 diagnostic long‐PCR (Figure S1). In a normal control, 12 kbp and 8 kbp PCR products were amplified with primer sets 1‐A/1‐B and 1‐P/1‐Q, respectively. In contrast, in a patient with HA and *F8* Inv1, 11 kbp and 9 kbp PCR products were amplified with a primer set 1‐A/1‐Q and 1‐B/1‐P, respectively. In the proband, the PCR using primer sets 1‐A/1‐B and 1‐A/1‐Q amplified products identical in size to 1‐A/1‐B in the normal control and to 1‐A/1‐Q in Inv1 as in the positive HA. However, the primer sets, 1‐P/1‐Q and 1‐B/1‐P, did not detect any amplicons. These observations indicated that the proband's X chromosome had an intact *int1h*‐2 (positive with primer set 1‐A/1‐B) and an inverted type of *int1h‐*1/2 (positive with primer set 1‐A/1‐Q).

Given the observations of the two breakpoints and a partial inversion structure in the proband's X chromosome, we hypothesized that the X chromosome possessed a rearranged structure with an intra‐chromosomal inversion and/or duplication (Figure 5a). In order to determine the abnormal structure of the patient's X chromosome, we performed a quantitative gene mapping analysis (Figure 5b). Copy number variations of *F8* and its neighboring loci were quantitatively examined. The patient's *F8* intron 1, *int1h*‐1, *int1h*‐2, and *VBP1* were in equal amounts compared to the ones in a pooled healthy male gDNA. In contrast, *FUNDC2* and *BRCC3* were assessed to have doubled gene dosage, indicating that a genomic sequence that ranged from a part of the *FUNDC2* to *BRCC3* was duplicated in the patient X chromosome.

Consequently, we suggested that the structure of the X chromosome in the proband was as follows: the patient *F8* was split with an over 119.5 kb of intra‐chromosomal inverted/duplicated alignment, described as NC_000023.11:g.155007148_155013478delins155028224_155147780inv (Figure 5c). The large inset broke the *F8* intron 1 via two unrelated breakpoints, the telomeric breakpoint with two base homology and the centromeric breakpoint associated with recombination of *int1h*‐1/2. This recombination resulted in a deletion of 6.3 kb alignment (NC_000023.11 nt155,007,148 to 155,013,480) in the *F8* intron 1.

## 4 DISCUSSION

In this study, we investigated the X chromosome structure in a patient with severe HA and demonstrated the complex genomic rearrangement with a split *F8* due to a 119.5 kb duplicated insertion in the *F8* intron 1. The telomeric breakpoint was a linkage of the inverted *FUNDC2* (nt155,028,224) to the *F8* intron 1 (nt155,013,481) via two bases of microhomology (Figure 3). The centromeric breakpoint was a recombination of *int1h*‐1 and ‐2 (Figure 4).

Several studies have pointed out unusual patterns of the *F8* Inv1 in patients with severe HA. Pio et al. (Pio et al., 2011) and Stefanovska et al. reported the case of a patient with severe HA who had intact and inverted types of *int1h*s (Sukarova Stefanovska et al., 2008). Sanna et al. reported the case of a patient with severe HA who carried a structural rearrangement with both normal and inverted *int1h*‐1 with 19.32 kb of duplication spanning the *F8* exon 2 to exon 6 (Sanna et al., 2013). Moreover, You et al. reported a mutated X chromosome with an extragenic segmental duplicon of 227.3 kb and a deletion in the *F8* intron 1 of 2.56 kb (You et al., 2014).

**Figure 6:**
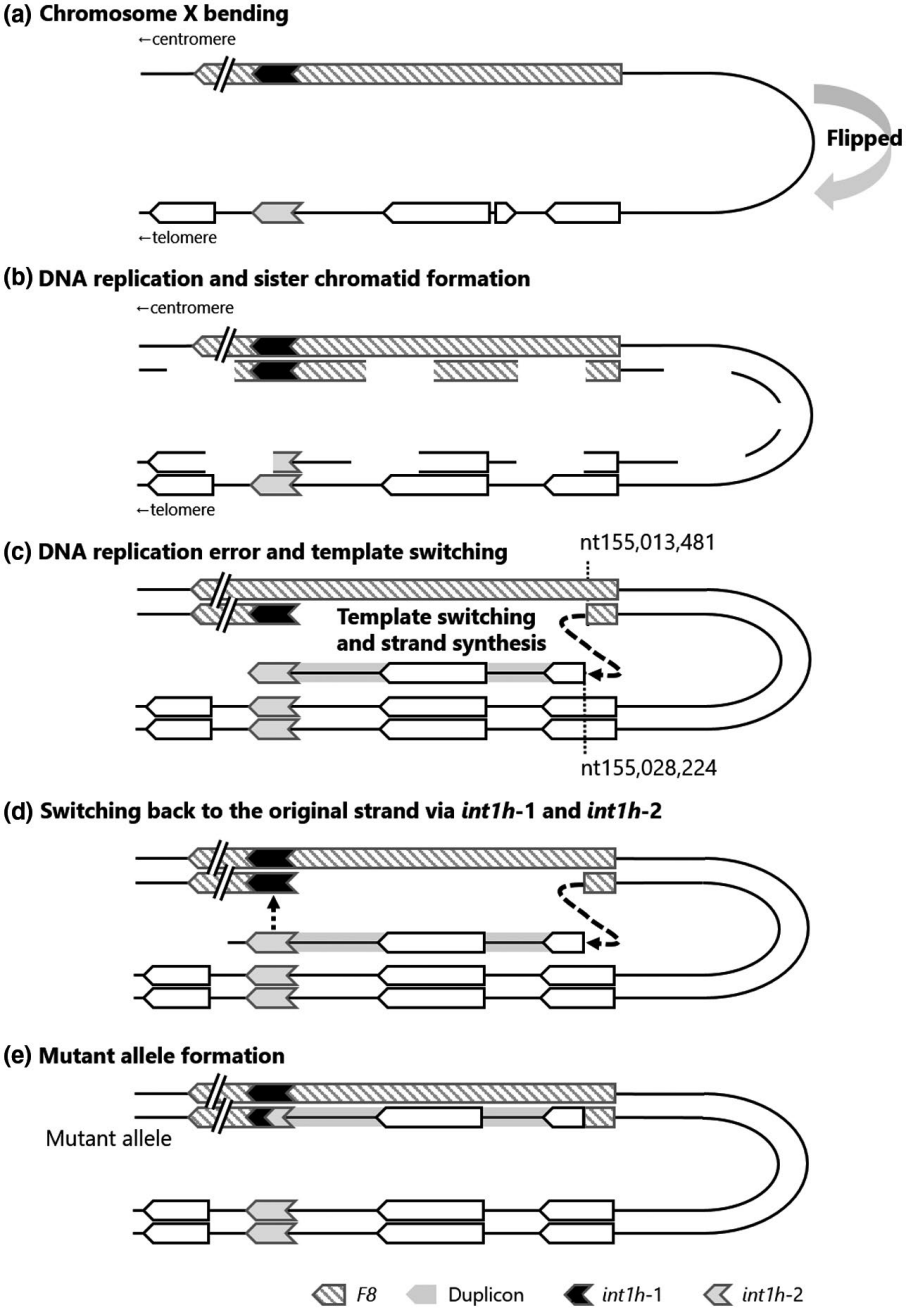
Hypothetical multi‐step mechanism forming the abnormal chromosome X structure. (a) Chromosome X bending. A telomeric end of chromosome X is flipped prior to DNA replication of chromosome X. (b and c) Template switching during DNA replication. A replication error occurred in a DNA replication fork at NC_000023.11 nt155,013,481 (*F8* intron 1) followed by an intra‐chromosomal template switching into NC_000023.11 nt155,028,224 (*FUNDC2*) with two bases of microhomology. This strand switching could be explained by FoSTeS or MMBIR. (d and e) Template switching back and mutant allele formation. After synthesizing a duplicon strand, the abnormal strand switched back to the original strand via an *int1h*‐1 on the original strand and an *int1h*‐2 on the abnormal strand. The recombination machinery between the *int1h*‐1 and ‐2 could have occurred due to homologous recombination or FoSTeS/MMBIR

In order to explain the structural abnormality of the proband's X chromosome, we propose a multi‐step rearrangement mechanism (Figure 6). In this model, a telomeric end of the X chromosome is flipped prior to DNA replication (Figure 6a). During DNA replication and sister chromatid formation, a replication error occurs in the DNA replication fork at NC_000023.11 nt155,013,481 (*F8* intron 1) followed by an intra‐chromosomal template switching into NC_000023.11 nt155,028,224 (*FUNDC2*; Figure 6b,c) with two bases of microhomology that forms the telomeric breakpoint. This strand switching may be explained using FoSTeS or MMBIR (Hastings, Ira, & Lupski, 2009; Lee, Carvalho, & Lupski, 2007). After synthesizing a duplicon strand, the abnormal strand switches back to the original strand via an *int1h*‐1 on the original strand and an *int1h*‐2 on the abnormal strand (Figure 6d,e). The recombination machinery between the *int1h*‐1 and ‐2 might have occurred because of a HR or a template switching. In agreement with the present model, a few reports also mentioned that similar chromosomal rearrangements associated with *F8* possibly resulted from a combination with the FoSTeS/MMBIR and HR (Chen et al., 2017; Sanna et al., 2013; You et al., 2014).

The proband's mother somatically carries the same abnormal X chromosome, which suggests two conceivable periods of rearrangement occurrence: an early stage of the meiotic phase in either maternal grandparents or an early embryogenesis of the proband's mother. Considering that a recurrent X chromosome inversion, such as *F8* Inv22, frequently occurs more in male meiosis than in female meiosis (Rossiter et al., 1994), the present rearrangement might have occurred during spermatogenesis in the maternal grandfather. In contrast, Chen et al described that the *F8* pathogenic variant due to a complex recombination mediated by sequential MMBIR and BIR may occur during an early period of embryogenesis in the proband's mother, which results in a somatic mosaicism (Chen et al., 2017). However, in the present study, since we only obtained gDNA extracted from peripheral blood cells from the patient, we were not able to investigate whether the proband's mother possessed a mosaicism with aberrant X chromosome.

In addition to the genomic investigations, we also analyzed *F8* mRNA in the proband (Figure S5). FVIII is synthesized in the liver sinusoidal endothelial cells (Do, Healey, Waller, & Lollar, 1999; Pan et al., 2016). In contrast, it is known that *F8* mRNA is expressed in PBMCs (Naylor, Green, Montandon, Rizza, & Giannelli, 1991), and the *F8* mRNA in PBMCs has been used to analyze a transcript abnormality in patients with HA (Chelly, Concordet, Kaplan, & Kahn, 1989; El‐Maarri et al., 2005; Naylor et al., 1991). We therefore analyzed the *F8* transcript in PBMCs from the proband. However, no *F8* transcript and not even a truncated fragment that might have been responsible for the proband's FVIII:Ag null phenotype were observed. It is possible that the inverted duplication insertion created novel splicing sites leading to aberrant transcripts that were rapidly degraded by mRNA decay mechanisms such as nonsense‐mediated mRNA decay (Byers, 2002).

In conclusion, we identified the complex abnormal X chromosome in the patient with severe HA, described as NC_000023.11:g.155007148_155013478delins155028224_155147780inv. The 119.5 kb duplicon derived from the *F8* extragenic alignment was inserted into the *F8* intron 1 with two breakpoints, resulting in thorough splitting of *F8*. We considered that the aberrant X chromosome was constructed through multi‐step rearrangement with FoSTeS/MMBIR and the *int1h*‐1/2‐associated recombination during a sister chromatid formation in a spermatogenesis of the maternal grandfather or an early embryogenesis of the proband's mother. This study provides new insights into the mechanisms responsible for several genomic repair machineries involved in deleterious *F8* rearrangement and contributes to precise genetic diagnostics in patients with HA.

## CONFLICT OF INTEREST

The authors declare that there is no conflict of interest regarding the publication of this manuscript.

## AUTHOR CONTRIBUTION

M. T. and S. T. designed and performed the research, analyzed data, and drafted the manuscript. N. S. designed the project, collected, and analyzed the clinical data. M. K., Y. H., K. O., and S. S. conducted the research and analyzed data. A.T. developed and supervised the project. A. K., F. H., S. O., A. S., T. Kanematsu., and T. M. developed the project and collected and analyzed the clinical data. T. Kojima designed the project, analyzed data, and drafted the manuscript.

## Supporting information

Figure S1‐S5

Table S1‐S4

## Data Availability

The data that support the findings of this study are available from the corresponding author upon reasonable request.
