# Supplementary material for: Aberrant X chromosomal rearrangement through multi‐step template switching during sister chromatid formation in a patient with severe hemophilia A
Source: Mol Genet Genomic Med. 2020 Jul 5;8(9):e1390. doi: 10.1002/mgg3.1390 (PMC7507428; doi:10.1002/mgg3.1390)
Supplement: Supplementary file 1 — Figure S1‐S5 [file MGG3-8-e1390-s001.pdf]

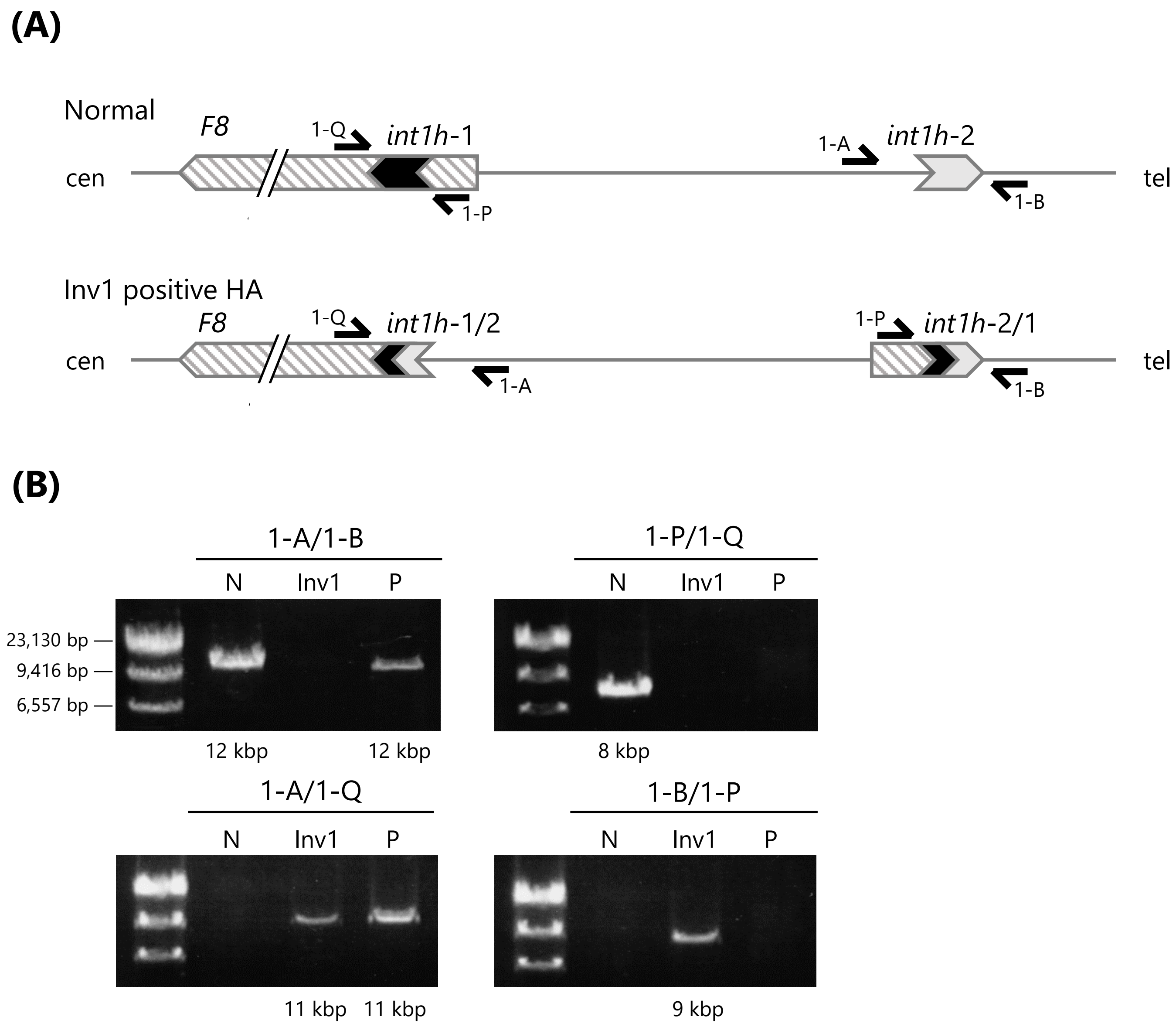

Supplementary Fig. 1

F8 Inv1 diagnostic Long-PCR. (A) PCR primer design. In a normal control, 12 kbp and 8 kbp of PCR products were amplified with primer sets 1-A/1-B and 1-P/1-Q, respectively. In a HA patient with *F8* Inv1, 11 kbp and 9 kbp of PCR products were amplified by primer sets 1-A/1-Q and 1-B/1-P, respectively. (B) Results of the proband's gDNA. The primer sets 1-A/1-B and 1-A/1-Q amplified their products; however, the primer sets, 1-P/1-Q and 1-B/1-P, did not detect any amplicons. N, normal male control; Inv1, HA patient carrying Inv1; P, proband.

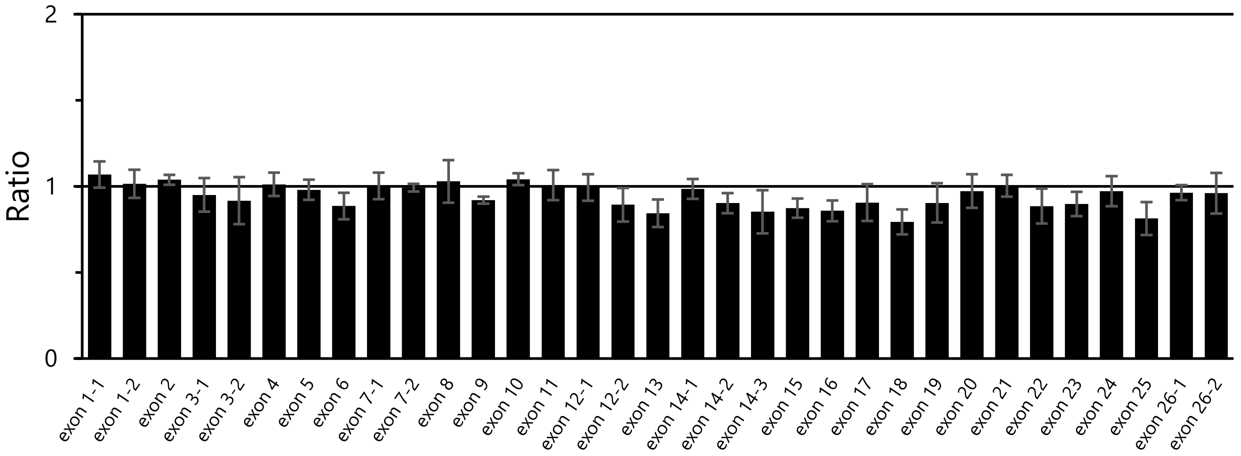

Supplementary Fig. 2

Copy number analysis of *F8* exons. To investigate an exonic deletion or duplication, MLPA was carried out. Gene dosage was normalized to a gDNA sample pooled with three normal male subjects. The proband's gDNA did not represent any copy number alteration in *F8* exons.

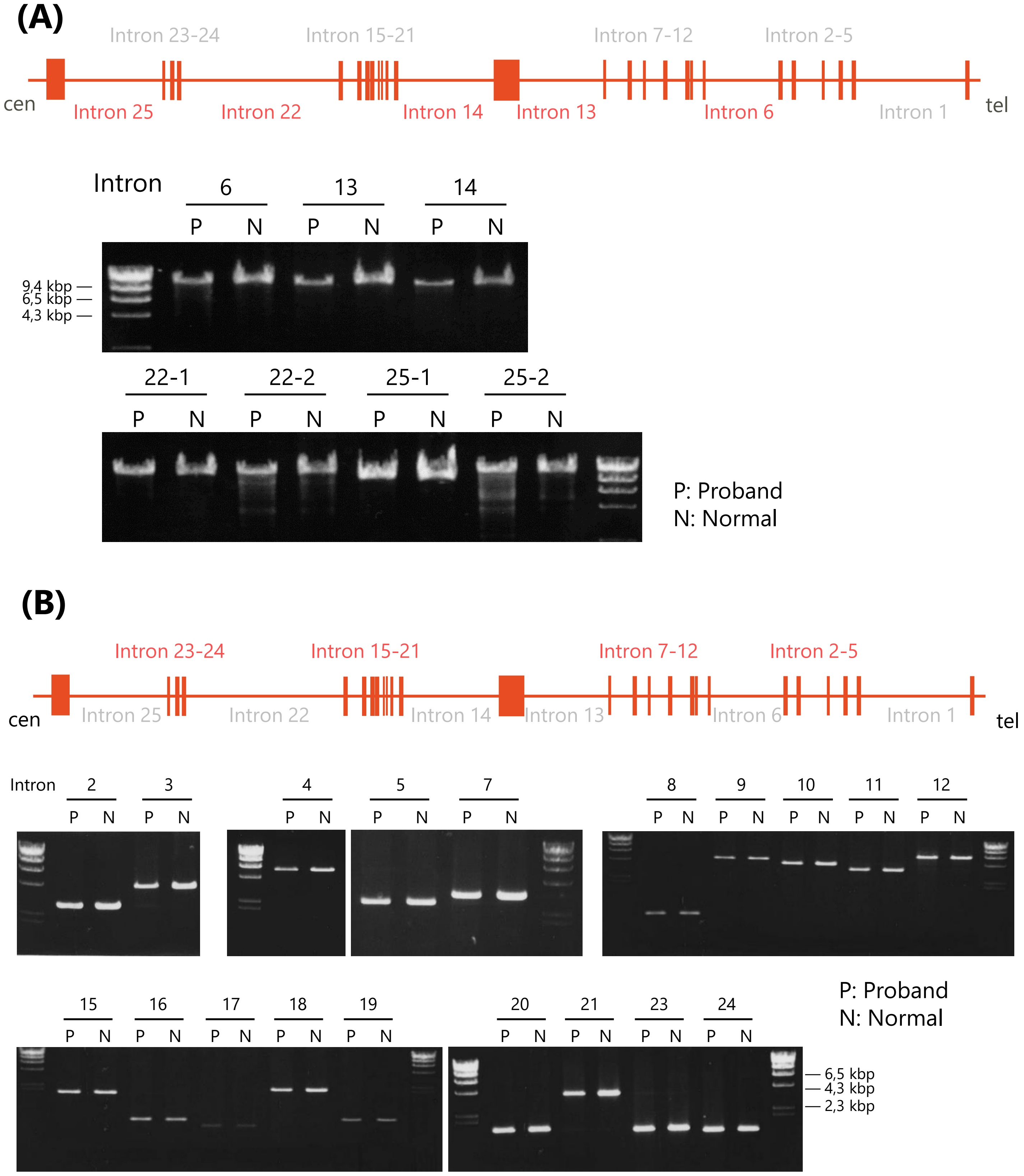

Supplementary Fig. 3

PCR mapping of *F8* introns, except for intron 1. (A) Screening results of large introns: intron 6, 13, 14, 22, and 25. (B) Screening results of small introns: intron 2, 3, 4, 5, 7, 8, 9, 10, 11, 12, 15, 16, 17, 18, 19, 20, 21, 23, and 24.

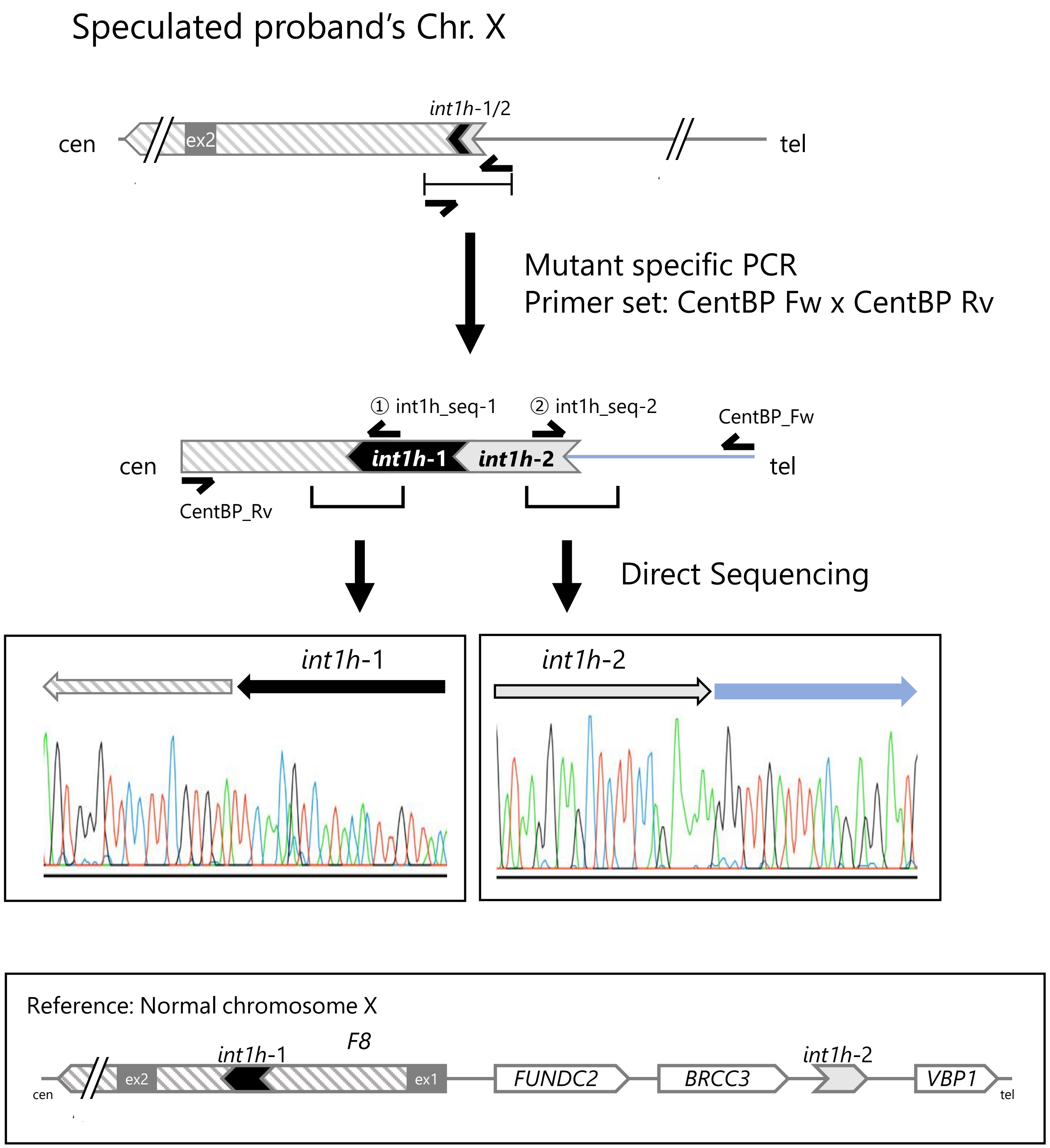

Supplementary Fig. 4

Direct sequence of mutant specific PCR product. The mutant specific product was subjected to a direct sequencing with a sequencing primer, int1h_seq-1 (circled 1) or int1h_seq-2 (circled 2), which revealed that mutant chromosome X possessed an inversion structure with a recombination between *F8 int1h*-1 and 2.

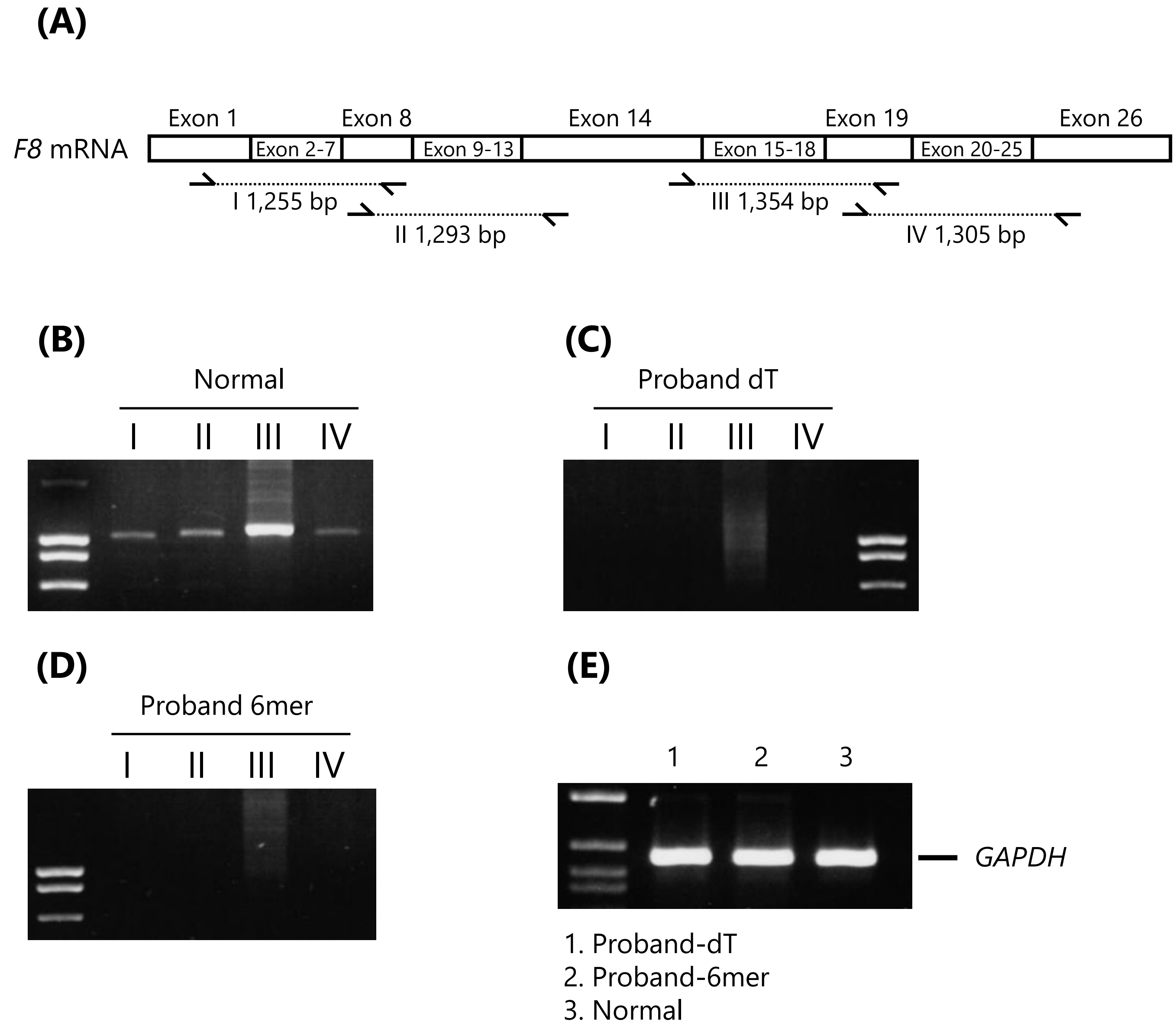

Supplementary Fig. 5

RT-PCR analysis of an ectopic *F8* mRNA isolated from peripheral blood mononuclear cells. (A) RT-PCR primer design detecting a *F8* mRNA. In this design, *F8* mRNA is divided into 4 segments, exon 1 to 8 (I), exon 8 to 14 (II), exon 14 to 19 (III), and exon 19 to 26 (IV). (C, D) RT-PCR in a proband's cDNA synthesized with oligo dT primer (C) or random 6 mer primer (D). (E) *GAPDH* as RT-PCR validating internal control.
