# Supplementary material for: Aberrant X chromosomal rearrangement through multi‐step template switching during sister chromatid formation in a patient with severe hemophilia A
Source: Mol Genet Genomic Med. 2020 Jul 5;8(9):e1390. doi: 10.1002/mgg3.1390 (PMC7507428; doi:10.1002/mgg3.1390)
Supplement: Supplementary file 2 — Table S1‐S4 [file MGG3-8-e1390-s002.pdf]

Supplementary table 1. Sequence of primers for *F8* recurrent inversion analysis. Location are coresponding to the NCBI nucleotide reference sequence NC_000023.11 in GRCh38.p13 (accessed on March 12, 2020).

Primer name	Sequence(5'-3')	Location		Application	Remarks column	Reference
		5' position	3' position			
ID	TTC CTA GCA CAT ACG GTT TAG TCA CAA G	154,877,146	154,877,119	IS-PCR (Inv 22 test)		Rossetti, Radic, Larripa, & De Brasi, 2008
ED	TTT TCC CCC AGT CAC TTA GGC TCA	155,466,204	155,466,227			
		155,374,510	155,374,487			
1U	CCC CCT TTC AAC TCC ATC TCC AT	154,898,257	154,898,279			
2U	CTT TAC TGA ACT TGT TTA TCA AAT CTA CGT GTC	155,387,905	155,387,937			
3U	CTC ACA TTG TGT TCT TGT AGT CAG AGT GTA CT	155,450,592	155,450,561			
1-ID	CTT TTC TTG AGT CTG CAA CTG GTA CTC ATC	155,005,518	155,005,489	IS-PCR (Inv1 test)		
1-ED	GAT CTG CTT CTC TTT CTG TGT ACC CTT C	155,148,927	155,148,954			
1-IU	GGC CGA TTG CTT ATT TAT ATC TCC AAG	155,018,164	155,018,190			
1-A	AAG GTA CCA GCC CAG GAA GAC ATC ATA CTC	155,144,967	155,144,996	Inv1 diagnostic long-PCR		
1-B	CCT TTG GGA AGC ATA TCA TCC TGC GAA GAG	155,156,742	155,156,713			
1-P	GTC CAC TCA CTA ATA TGC CTT TGT CCC AAC	155,007,178	155,007,149			
1-Q	TAT TTG ACA TCT AAG TGG AGG TAA GCA GTT GG	154,999,253	155,999,284			

Supplementary table 2. Sequence of primers for *F8* DNA sequencing. Location are coresponding to the NCBI nucleotide reference sequence NC_000023.11 in GRCh38.p13 (accessed on March 12, 2020).

Primer name	Sequence(5'-3')		Location	
	N13 U/L	Gene specific	5' position	3' position
F8 P3U	gta gcg cga cgg cca gt	GAG CTC ACC ATG GCT ACA TTC	155,023,727	155,023,707
F8 P3L	cag ggc gca gcg atg ac	CAT GGT CTG CAT GCC CTT T	155,023,287	155,023,305
F8 P2U	gta gcg cga cgg cca gt	TTA GGG AAG AGT AAG GAG ACC AG	155,023,365	155,023,343
F8 P2L	cag ggc gca gcg atg ac	AGT GGC AGC AGC AAG AGA AG	155,022,954	155,022,973
F8 P1U	gta gcg cga cgg cca gt	AGG TTG CTG GTT ACT CTT GCT A	155,023,033	155,023,012
F8 P1L	cag ggc gca gcg atg ac	TGC AGA GCA TTT TAA GGA ACT TT	155,022,674	155,022,696
F8 1U	gta gcg cga cgg cca gt	TAG CAG CCT CCC TTT TGC TA	155,022,803	155,022,784
F8 1L	cag ggc gca gcg atg ac	CTA ACC CGA TGT CTG CAC CT	155,022,324	155,022,343
F8 2U	gta gcg cga cgg cca gt	CAT TAC TTC CAG CTG CTT TTT G	154,999,665	154,999,644
F8 2L	cag ggc gca gcg atg ac	TTT GGC AGC TGC ACT TTT TA	154,999,376	154,999,395
F8 3U	gta gcg cga cgg cca gt	GCA TGC TTC TCC ACT GTG AC	154,997,195	154,997,176
F8 3L	cag ggc gca gcg atg ac	GCC ACC ATT ACA AAG CAC AC	154,996,897	154,996,916
F8 4U	gta gcg cga cgg cca gt	CAT GTT TCT TTG AGT GTA CAG TGG	154,993,208	154,993,185
F8 4L	cag ggc gca gcg atg ac	TTC AGG TGA AGG AAC ACA AAT G	154,992,837	154,992,858
F8 5U	gta gcg cga cgg cca gt	TCT CCT CCT AGT GAC AAT TTC C	154,987,377	154,987,356
F8 5L	cag ggc gca gcg atg ac	CCC ATC TCC TTC ATT CCT GA	154,987,119	154,987,138
F8 6U	gta gcg cga cgg cca gt	GCG GTC ATT CAT GAG ACA CA	154,984,861	154,984,842
F8 6L	cag ggc gca gcg atg ac	CCG AGC TGT TTG TGA ACT GA	154,984,604	154,984,623
F8 7U	gta gcg cga cgg cca gt	TGT CCT AGC AAG TGT TTT CCA TT	154,969,623	154,969,601
F8 7L	cag ggc gca gcg atg ac	AAT GTC CCC TTC AGC AAC AC	154,969,224	154,969,243
F8 8U	gta gcg cga cgg cca gt	CAC CAT GCT TCC CAT ATA GC	154,966,821	154,966,802
F8 8L	cag ggc gca gcg atg ac	ATG GCT TCA GGA TTT GTT GG	154,966,338	154,966,357
F8 9U	gta gcg cga cgg cca gt	TTT GAG CCT ACC TAG AAT TTT TCT TC	154,966,198	154,966,173
F8 9L	cag ggc gca gcg atg ac	GGT ATT TTA GAA ACT CAA AAC TCT CC	154,965,899	154,965,924
F8 10U	gta gcg cga cgg cca gt	TTC TTG TTG ATC CTA GTC GTT TT	154,961,225	154,961,203
F8 10L	cag ggc gca gcg atg ac	GCT GGA GAA AGG ACC AAC ATA	154,960,976	154,960,996
F8 11U	gta gcg cga cgg cca gt	CCC TTG CAA CAA CAA CAT GA	154,957,251	154,957,232
F8 11L	cag ggc gca gcg atg ac	TTT CTT CAG GTT ATA AGG GGA CA	154,956,890	154,956,912
F8 12U	gta gcg cga cgg cca gt	TGC TAG CTC CTA CCT GAC AAC A	154,954,107	154,954,086
F8 12L	cag ggc gca gcg atg ac	CAT TCA TTA TCT GGA CAT CAC TTT G	154,953,810	154,953,834
F8 13U	gta gcg cga cgg cca gt	CAT GAC AAT CAC AAT CCA AAA TA	154,947,977	154,947,977
F8 13L	cag ggc gca gcg atg ac	CAT GTG AGC TAG TGG GCA AA	154,947,614	154,947,633
F8 14A U	gta gcg cga cgg cca gt	ATC TGT GTT ATG AGT AAC CA	154,931,721	154,931,702
F8 14A L	cag ggc gca gcg atg ac	TTA TTA CTG TCT ATT GCT CC	154,931,247	154,931,266
F8 14B U	gta gcg cga cgg cca gt	CAT GGG CTA TCC TTA TCT GA	154,931,332	154,931,313
F8 14B L	cag ggc gca gcg atg ac	CAT GAA CTT TCT TGG CTA TT	154,930,854	154,930,873
F8 14C U	gta gcg cga cgg cca gt	TCA AAG TTG TTA GAA TCA GG	154,930,900	154,930,881
F8 14C L	cag ggc gca gcg atg ac	ATT TTG TGC ATC TGG TGG AA	154,930,460	154,930,479
F8 14D U	gta gcg cga cgg cca gt	GTC CAA CAG AAA AAA GAG GG	154,930,504	154,930,485
F8 14D L	cag ggc gca gcg atg ac	CTA CAT TTT GCC TAG TGC TC	154,930,024	154,930,043
F8 14E U	gta gcg cga cgg cca gt	CTG GCA CTA AGA ATT TCA TG	154,930,080	154,930,061
F8 14E L	cag ggc gca gcg atg ac	CCT TCT CAT TGT AGT CTA TC	154,929,652	154,929,671
F8 14F U	gta gcg cga cgg cca gt	GAA ACA TTT GAC CCC GAG CA	154,929,701	154,929,682
F8 14F L	cag ggc gca gcg atg ac	TTT TGG GCA AGT CTG GTT TC	154,929,271	154,929,790
F8 14G U	gta gcg cga cgg cca gt	CAC ATA CAA GAA AGT TGA GA	154,929,323	154,929,304
F8 14G L	cag ggc gca gcg atg ac	CTC ATT TAT TGC TGC TAT TG	154,928,888	154,928,907
F8 14H U	gta gcg cga cgg cca gt	GAT ACC ATT TTG TCC CTG AA	154,928,947	154,928,928
F8 14H L	cag ggc gca gcg atg ac	GTC ACA AGA GCA GAG CAA AG	154,928,530	154,928,549
F8 15U	gta gcg cga cgg cca gt	TGA GGC ATT TCT ACC CAC TTG	154,906,635	154,906,615
F8 15L	cag ggc gca gcg atg ac	CCA AAA GTG GGA ATA CAT TAT AGT CA	154,906,337	154,906,362
F8 16U	gta gcg cga cgg cca gt	CAG CAT CCA TCT TCT GTA CCA	154,905,177	154,905,157
F8 16L	cag ggc gca gcg atg ac	AAA GCT TCT TAT TGC ACG TAG G	154,904,710	154,904,731
F8 17U	gta gcg cga cgg cca gt	AGG TTG GAC TGG CAT AAA AA	154,904,616	154,904,597
F8 17L	cag ggc gca gcg atg ac	CCC TGG ATC AAG TCT CAT TTG	154,904,221	154,904,241
F8 18U	gta gcg cga cgg cca gt	TGG TGG AGT GGA GAG AAA GAA	154,904,154	154,904,134
F8 18L	cag ggc gca gcg atg ac	AGC ATG GAG CTT GTC TGC TT	154,903,812	154,903,793
F8 19U	gta gcg cga cgg cca gt	AAC CAA TGT ATC TCA TGC TCA TTT T	154,902,235	154,902,211
F8 19L	cag ggc gca gcg atg ac	GGA AGA AAG CTG TAA AGA AGT AGG C	154,901,988	154,902,012
F8 20U	gta gcg cga cgg cca gt	TTT GAG AAG CTG AAT TTT GTG C	154,901,500	154,901,479
F8 20L	cag ggc gca gcg atg ac	GAA GCA TGG AGA TGG ATT CAT TA	154,901,272	154,901,294
F8 21U	gta gcg cga cgg cca gt	CCA CAG CTT AGA TTA ACC TTT CTC A	154,900,051	154,900,027
F8 21L	cag ggc gca gcg atg ac	TGA GCT TGC AAG AGG AAT AAG TAA	154,899,791	154,899,814
F8 22U	gta gcg cga cgg cca gt	TCA GGA GGT AGC ACA TAC AT	154,896,292	154,896,273
F8 22L	cag ggc gca gcg atg ac	GTC CAA TAT CTG AAA TCT GC	154,896,006	154,896,025
F8 23U	gta gcg cga cgg cca gt	TTG ACA GAA ATT GCT TTT TAC TCT G	154,863,292	154,863,268
F8 23L	cag ggc gca gcg atg ac	TCC CCC AGT CTC AGG ATA ACT	154,862,999	154,863,019
F8 24U	gta gcg cga cgg cca gt	ACT GAG GCT GAA GCA TGT CC	154,861,918	154,861,899
F8 24L	cag ggc gca gcg atg ac	CCC AAC CAC TGC TCT GAG TC	154,861,669	154,861,688
F8 25U	gta gcg cga cgg cca gt	TGG GAA TTT CTG GGA GTA AAT G	154,860,669	154,860,648
F8 25L	cag ggc gca gcg atg ac	AAG CTC TAG GAG AGG TGG TAT TTT T	154,860,370	154,860,394
F8 26U	gta gcg cga cgg cca gt	CTG TGC TTT GCA GTG ACC AT	154,837,815	154,837,796
F8 26L	cag ggc gca gcg atg ac	TGG AAG GAA GGA GTA ATC TGG	154,837,357	154,837,377
F8 UTR 1U	gta gcg cga cgg cca gt	AGG GTG CAT CCA ATT TAA CTT A	154,837,426	154,837,405
F8 UTR 1L	cag ggc gca gcg atg ac	GTG CCC CTC ATA ATG ACT AAA TAA	154,836,983	154,837,006
F8 UTR 2U	gta gcg cga cgg cca gt	TCT GCA AAA TGG AGA GAA TA	154,837,082	154,837,063
F8 UTR 2L	cag ggc gca gcg atg ac	GGA AAT TAG TAG AGG GAG AGA G	154,836,583	154,836,604
F8 UTR 3U	gta gcg cga cgg cca gt	ATG ATG ATG ACA TTA GGC TTC T	154,836,728	154,836,707
F8 UTR 3L	cag ggc gca gcg atg ac	TCC AAC TGC TCT ATA ACT TAT CC	154,836,279	154,836,301
F8 UTR 4U	gta gcg cga cgg cca gt	ACT ATG ACT ACA CAG AAT CTC CT	154,836,348	154,836,326
F8 UTR 4L	cag ggc gca gcg atg ac	AGA GAT GTA TAT AGT CAA TGG GA	154,835,879	154,835,901
F8 UTR 5U	gta gcg cga cgg cca gt	CCC CAA AGG TGA TAT GGT TTT	154,835,967	154,835,947
F8 UTR 5L	cag ggc gca gcg atg ac	TCA GTG TTC ACA TTT TTA TTT CCA	154,835,738	154,835,761
N13 U	gta gcg cga cgg cca gt			
N13 L	cag ggc gca gcg atg ac			

Supplementary table 3. Sequence of primers used to detect the breakpoints. Location are coresponding to the NCBI nucleotide reference sequence NC_000023.11 in GRCh38.p13 (accessed on March 12, 2020).

Primer name	Sequence(5'-3')	Location		Application	Remarks column	Reference
		5' position	3' position			
seg1_Fw	TTG GAG TAG GCT AGG AAT AGG AGC ACA AAT TA	155,018,187	155,018,158	*F8* intron 1 mapping PCR		
seg1_Rv	GGC CGA TTG CTT ATT TAT ATC TCC AAG	155,018,164	155,018,190		Same as 1-IU	
seg2_Fw	GGA GAT ATA AAT AAG CAA TCG GCC AGG TAT	155,018,187	155,018,158			
seg2_Rv	TAT GTG ATT CAG GAC AGG ACT CTA TTC AAA GG	155,009,965	155,009,996			
seg3_Fw	AAC CAC AGG TCA GGG AAT ATA ATA GGT TTT CC	155,010,381	155,010,350			
seg3_Rv	GAT GAG TAC CAG TTG CAG ACT CAA GAA AAG	155,005,489	155,005,518			
seg4_Fw	CTT TTC TTG AGT CTG CAA CTG GTA CTC ATC	155,005,518	155,005,489		Same as 1-ID	
seg4_Rv	TAT TTG ACA TCT AAG TGG AGG TAA GCA GTT GG	154,999,253	155,999,284		Same as 1-Q	
InvPCR_Fw	AAC AGT TCA ACT ATG CTA GCT CCA TAT ACC TT	155,018,309	155,018,278	*Pst* I-inverse PCR,<br>centromric mutant specific PCR		
InvPCR_Rv	AGT ACA TAG AAC CCA CTA ATG ATC AAA TTC CAT GA	155,019,011	155,019,045	*Pst* I-inverse PCR		
InvPCR_Rv2	ACT GTG CTG TAG CCT GAA TG	155,028,382	155,028,363	centromric mutant specific PCR		
BP_seq_Fw	GCG TCT GTG TTC ATG ATG GA	155,013,625	155,013,606	Sequencing of centromeric breakpoint		
InvPCR2_Fw	AAG TCC CTG AGA GTC AAT AAG ACA TGC GA	155,000,810	155,000,773	*Hin* dIII-inverse PCR		
InvPCR2_Rv	AGA ATG CTG AGA TAG AGA AGG AAA ATG CAA T	155,001,176	155,001,206			
CentBP_Fw	GTC CAC TCA CTA ATA TGC CTT TGT CCC AAC	155,007,178	155,007,149	Telomeric mutant specific PCR	Same as 1-P	
CentBP_Rv	AGA ATG CTG AGA TAG AGA AGG AAA ATG CAA T	155,028,382	155,028,363		Same as InvPCR2_Rv	
Int1h_seq-1	CTG GCA ATG AAT TCT CCC AA	155,148,749	155,148,768	Sequencing of telomeric breakpoint		
		155,006,179	155,006,160			
Int1h_seq-2	CCG AGA AAG CAC GTA GTC CA	155,147,898	155,147,879			
		155,007,030	155,007,049			

Supplementary table 4. Sequence of primers for quantitative gene mapping (real-time PCR) and *F8* mRNA RT-PCR. Location are coresponding to the NCBI nucleotide reference sequence NC_000023.11 in GRCh38.p13 (accessed on March 12, 2020).

Primer name	Sequence(5'-3')	Location		Application	Remarks column	Reference
		5' position	3' position			
F8_Intron1-1_Fw	AAC TGT GCT GCT TTA TTG GC	155,018,433	155,018,414	Quantitative gene mapping (*F8* intron 1-1)		
F8_Intron1-1_Rv	TGT TCA TGT TTT CCC ATC AGA C	155,018,334	155,018,355			
F8_Intron1-2_Fw	CCT TAA TTA CCC AAA TGC CCA T	155,000,563	155,000,542	Quantitative gene mapping (*F8* intron 1-2)		
F8_Intron1-2_Rv	CTG ACT TTT ATG CTT CTG GAC A	155,000,454	155,000,475			
int1h_Fw	GGA AAT GTG ATG TTT GGA CTG C	155,148,467	155,148,446	Quantitative gene mapping (*int1h*-1, -2)		
		155,006,461	155,006,482			
int1h_Rv	TTT CGA TCA TTC CCT GTG GT	155,148,340	155,148,359			
		155,006,588	155,006,569			
FUNDC2_Intron1_Fw	TCT GTC ATA GTG CTT AGC AGT	155,028,264	155,028,284	Quantitative gene mapping (*FUNDC2*)		
FUNDC2_Intron1_Rv	GGA AGG TAG ATT AAA TGC GAG A	155,028,330	155,028,309			
BRCC3_Intron1_Fw	AGG TGA GTA GGT CTG TTA GC	155,071,649	155,071,668	Quantitative gene mapping (*BRCC3*)		
BRCC3_Intron1_Rv	AGA GCT ATG TAA AGG GTG TCC A	155,071,789	155,071,768			
VBP1_Exon4_Fw	CTG GCC TGT TTA TGG TAC TGT C	155,228,341	155,228,362	Quantitative gene mapping (*VBP1*)		
VBP1_Exon4_Rv	GGT AGG AGG AAC TGA AGC TTT G	155,228,458	155,228,437			
RT-I_Fw	CTT CTC CAG TTG AAC ATT TG	155,022,584	155,022,565	*F8* mRNA RT-PCR		El-Maarri et al., 2005
RT-I_Rv	TCA GCA GCA ATG TAA TGT AC	154,966,474	154,966,493			
RT-II_Fw	CAT GGA AGC TTA TGT CAA AGT A	154,966,683	154,966,662			
RT-II_Rv	CTA GGG TGT CTT GAA TTC TGG	154,931,484	154,931,504			
RT-III_Fw	GGG AAA TAA CTC GTA CTA CT	154,928,790	154,928,771			
RT-III_Rv	GAG ATG TAG AGG CTG GAG AA	154,896,153	154,896,172			
RT-IV_Fw	TGA GAC AGT GGA AAT GTT AC	154,902,160	154,902,141			
RT-IV_Rv	TTG CCT AGT TAT ATT GGA AGG A	154,837,343	154,837,364			
